# *Poecilia vivipara* Bloch & Schneider, 1801 (Cyprinodontiformes, Poeciliidae), a guppy in an oceanic archipelago: from where did it come?

**DOI:** 10.3897/zookeys.746.20960

**Published:** 2018-03-26

**Authors:** Waldir Miron Berbel-Filho, Luciano Freitas Barros-Neto, Ricardo Marques Dias, Liana Figueiredo Mendes, Carlos Augusto Assumpção Figueiredo, Rodrigo Augusto Torres, Sergio Maia Queiroz Lima

**Affiliations:** 1 Laboratório de Ictiologia Sistemática e Evolutiva, Departamento de Botânica e Zoologia, Universidade Federal do Rio Grande do Norte, Av. Senador Salgado Filho 3000, 56078-970, Natal, RN, Brazil; 2 Department of BioSciences, College of Science, Swansea University, SA2 8PP, Swansea, Wales (present address); 3 Museu Nacional do Rio de Janeiro/UFRJ, Setor de Ictiologia, Departamento de Vertebrados, Quinta da Boa vista, s/n, 20940-040, Rio de Janeiro, RJ, Brasil; 4 Laboratório do Oceano, Departamento de Ecologia, Av. Senador Salgado Filho 3000, 56078-970, Natal, RN, Brazil; 5 Universidade Federal do Estado do Rio de Janeiro, Instituto de Biociências, Departamento de Ciências do Ambiente. Av. Pasteur, 458, sala 512-F, 22290-240, Rio de Janeiro, RJ, Brazil; 6 Laboratório de Genômica Evolutiva e Ambiental, Departamento de Zoologia, Universidade Federal de Pernambuco, 50570-420, Recife, PE, Brazil

**Keywords:** Fernando de Noronha arquipelago, Human-mediated dispersal, Mitochondrial DNA, Mosquitofish, Mosquito larvae control, Natural dispersal

## Abstract

*Poecilia vivipara*, a small euryhaline guppy is reported at the Maceió River micro-basin in the Fernando de Noronha oceanic archipelago, northeast Brazil. However, the origin (human-mediated or natural dispersal) of this insular population is still controversial. The present study investigates how this population is phylogenetically related to the surrounding continental populations using the cytochrome oxidase I mitochondrial gene from eleven river basins in South America. Our phylogenetic reconstruction showed a clear geographical distribution arrangement of *P. vivipara* lineages. The Fernando de Noronha haplotype fell within the 'north' clade, closely related to a shared haplotype between the Paraíba do Norte and Potengi basins; the geographically closest continental drainages. Our phylogenetic reconstruction also showed highly divergent lineages, suggesting that *P. vivipara* may represent a species complex along its wide distribution. Regarding to the insular population, *P. vivipara* may have been intentionally introduced to the archipelago for the purpose of mosquito larvae control during the occupation of a U.S. military base following World War II. However, given the euryhaline capacity of *P. vivipara*, a potential scenario of natural (passive or active) dispersal cannot be ruled out.

## Introduction

The origin of terrestrial and freshwater organisms on oceanic islands has historically been a topic of intrigue within the field of biogeography. Oceanic islands are created by volcanic or coralline processes (de Queiroz 2005), making them isolated from the continent. As a result, these islands typically exhibit depauperate freshwater ichthyofauna, as such species are physiological incapable of dispersing across salt water (McDowall 2004). Thus, excluding introduced species, only secondary freshwater fishes (which can tolerate salinity and occasionally cross small marine barriers) or peripheral fishes (freshwater species with a recent marine origin) can transverse this biogeographic barrier and are naturally found on those oceanic islands (Bianco and Nordlie 2008; Walter et al. 2012).

There are four oceanic archipelagos in the Brazilian territory: Rocas Atoll, Fernando de Noronha, São Pedro and São Paulo, and Trindade and Martin Vaz (Serafini et al. 2010). The Fernando de Noronha archipelago is a Brazilian Protected Area composed of 21 volcanic islands, in an area of c. 26 km^2^ (Barcellos et al. 2011), located 345 km off the northeast Brazilian coast (Rangel and Mendes 2009). There are reports of freshwater fish species across the Fernando de Noronha archipelago, including species used as alternative food sources, such as the tambaqui *Colossoma macropomum* (Curvier 1816) and the tilapia *Oreochromis niloticus* (Linneaus, 1718) (Soto 2001; 2009). Other species, such as the guppy (or mosquitofish) *Poecilia vivipara* Bloch & Schneider, 1801, were supposedly introduced for mosquito larvae control. (Soto 2001; 2009).

Originally described from Suriname, *P. vivipara* is a small poeciliid species found mainly in lentic waters, ranging in salinity from freshwater to hypersaline conditions (Gomes-Jr and Monteiro 2007). The known distribution of *P. vivipara* spans from the delta of the Orinoco River (Venezuela) to Uruguay. The species may have potentially been introduced to Puerto Rico (Lucinda 2003) and Martinique (Lim et al. 2002), both of which are oceanic islands in the Caribbean Sea. It is thought that *P. vivipara* was intentionally introduced to the Fernando de Noronha archipelago (Brazil) to control mosquito larvae during the installation of the World War II military bases (Soto 2009). However, there are no studies investigating whether this is indeed an introduced or a native species, and therefore the full geographic range of this species remains unknown (Soto 2001).

Molecular approaches have been used to identify the source regions of introduced species, particularly in cases where the species in question has a wide geographical range and introduction events are not well documented (Roux and Wieczorek 2009). Given that *P. vivipara* is a continental euryhaline fish species, the present study aims to shed light onto the presence of *P. vivipara* in the Fernando de Noronha oceanic islands using phylogenetic analysis of mitochondrial DNA. The overall objective is to identify how this isolated population is phylogenetically related to its continental conspecifics.

## Materials and methods

**Figure 1.**
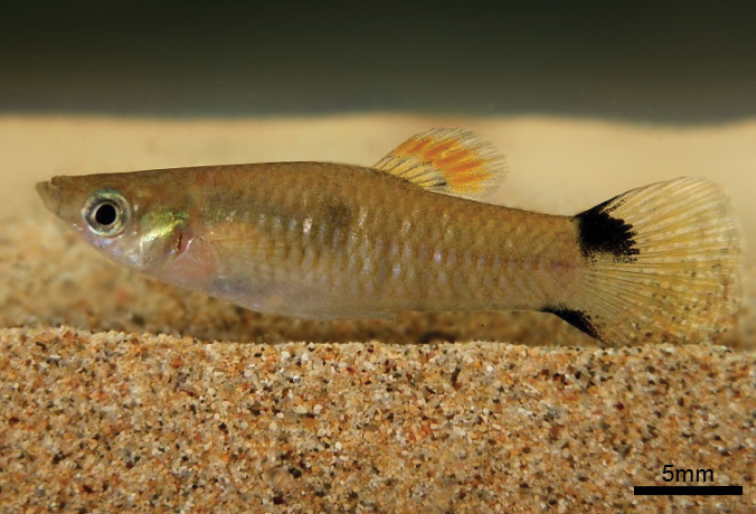
Live male of *Poecilia vivipara*, UFRN 0225, 25.2 mm SL. Maceió River microbasin, Fernando de Noronha Archipelago, Pernambuco, Brazil.

**Figure 2.**
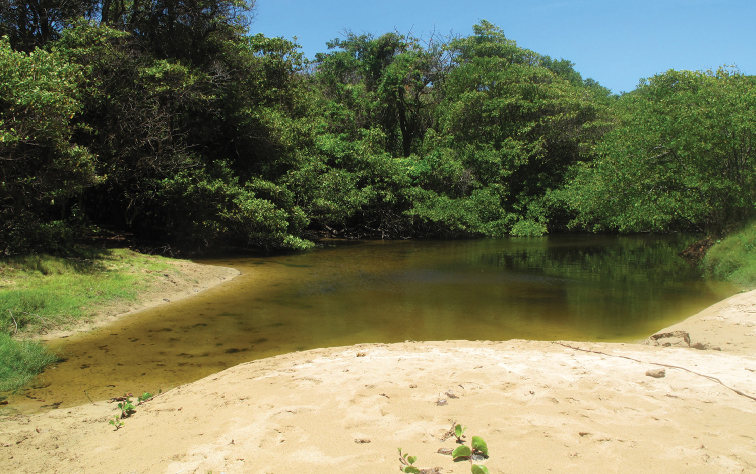
Sampling site of *Poecilia vivipara* in the border of the mangrove at Maceió River microbasin, Fernando de Noronha Archipelago, Pernambuco, Brazil.

During a field trip to sample *Bathygobius soporator* (Valenciennes 1837) at the Fernando de Noronha archipelago, we unexpectally found *P. vivipara* (Fig. 1). More specifically, in the estuary of the Maceió River micro basin (Fig. 2). A total of of 43 *P. vivipara* individuals (5 males and 38 females, 9.0–42.9 mm SL) were collected using hand nets and plastic bags. The site of species occurrence (03°51'57.80"S, 32°25'32.79"W) is the only oceanic mangrove in the South Atlantic (Serafini et al. 2010), and is comprised of a single mangrove tree species, *Laguncularia racemosa* (L.) Gaertn (Batistella 1996) (Fig. 2) covering an area ca. 0.01 km^2^. Five individuals were fixed and stored in 100 % ethanol for molecular analysis. These samples were deposited at the fish collection of the Universidade Federal do Rio Grande do Norte (UFRN 0225 and UFRN 0822). Sampling was conducted under an ICMBio/MMA permit (10806-4/2011).

DNA extraction was performed using the DNA easy Tissue Kit (Qiagen). Cytochrome Oxidase I (COI) mitochondrial DNA gene was amplified, using the primers FISH-BCH2 (5’ ACTTCYGGGTGRCCRAARAATCA 3’) and FISH-BCL (5’ TCAACYAATCAYAAAGATATYGGCAC 3’) (Tornabene et al. 2010). PCR reactions (25 µL of final volume) were performed using 10–30 ng of DNA template, 0.10 ng/uL of each primer, 12.5 µL of 2x Taq Master Mix Vivantis^M^, and 10.2 µL of ultrapure water. Amplification consisted of an initial cycle at 95 °C for 5 min, followed by 35 cycles of 94 °C for 30 s, 50 °C for 35 s, 72 °C for 70 s, a final extension step of 72 °C for 7 min, and 2 min at 20 °C. PCR product was examined using a1 % agarose gel and purified using the QIAquick PCR Purification Kit (Qiagen). All sequencing reactions were performed using Big Dye v3.1 (Applied Biosystems) and screened using ABI PRISM 3500 Genetic Analyzer (Applied Biosystems). All sequences obtained in this study were deposited in GenBank (Table 1).

**Table 1.** List of species, sampling sites (basin, municipality, state, country, and number in the map), geographical coordinates, catalogue number and GenBank access number used in the phylogenetic analysis. Abbreviations: N, number of individuals; *, sequences from GenBank; CE, Ceará State; PB, Paraíba State; MG, Minas Gerais State; PE, Pernambuco State; PR, Paraná State, RJ; Rio de Janeiro State; RN, Rio Grande do Norte State; SE, Sergipe State.

Species	N	Sampling site	Latitude	Longitude	Voucher N°	GenBank N°
Poecilia vivipara*	3	Macanao, Margarita island, Nueva Esparta, Venezuela (1)	10,988, -64,164		–	KP761881–KP761883
Poecilia vivipara*	2	Tubores, Margarita island, Nueva Esparta , Venezuela (1)	10,905, -64,107		–	KP761884–KP761885
*Poecilia vivipara*	4	Maceió, Fernando de Noronha, PE, Brazil (2)	-3,865, -32,425		UFRN0822	KU684422–KU684425
*Poecilia vivipara*	1	Jaguaribe, Saboeiro, CE, Brazil (3)	-6,541, -39,910		UFRN0337	KU684421
*Poecilia vivipara*	5	Piranhas-Açu, Serra Negra do Norte, RN, Brazil (4)	-6,579, -37,255		UFRN0289	KU684426–KU684430
*Poecilia vivipara*	4	Potengi, Macaíba, RN, Brazil (5)	-5,881, -35,369		UFRN2694	KU684417–KU684420
*Poecilia vivipara*	4	Paraíba do Norte, Barra de Santana, PB, Brazil (6)	-7,529, -35,998		UFRN0431	KU684431–KU684434
*Poecilia vivipara*	3	Ipojuca, Ipojuca, PE, Brazil (7)	-8,583, -35,043		UFRN1072	KU684414–KU684416
*Poecilia vivipara*	3	São Francisco, Serra Talhada, PE, Brazil (8)	-8,211, -38,534		UFRN0529	KU684441–KU684443
*Poecilia vivipara*	3	Piauí, Estância, SE, Brazil (9)	-11,209, -37,282		UFRN0823	KU684438–KU684440
*Poecilia vivipara*	3	São João, São João da Barra, RJ, Brazil (10)	-22,523, -42,559		UFRN1074	KU684435–KU684437
Poecilia vivipara*	1	Paraná, Piraguaçu, MG, Brazil (11)	-22,613, -45,514		–	GU701911
Poecilia vivipara*	2	Paraná, Califórnia, PR, Brazil (11)	-23,675, -51,313		–	GU70190; GU701908
Poecilia vivipara*	1	Paraná, Campo Mourão, PR, Brazil (11)	-24,078, -52,296		–	GU701904
Poecilia hondurensis*	2	Aguán, Honduras	–	–	–	JX968669–JX968670
Poecilia mexicana*	1	Lempa, El Salvador	–	–	–	JX968662– JX968663
Poecilia reticulata*	2	Pernadeles, Dominican Replubic	–	–	–	X968695–X968696
Poecilia sphenops*	2	Nahualate, Guatemala	–	–	–	JX968660–JX968661
*Pamphorichthyshollandi*	4	Sergipe, Aracaju, SE, Brazil	-10,926, -37,102		UFRN3663	KU484444–KU684447

**Figure 3.**
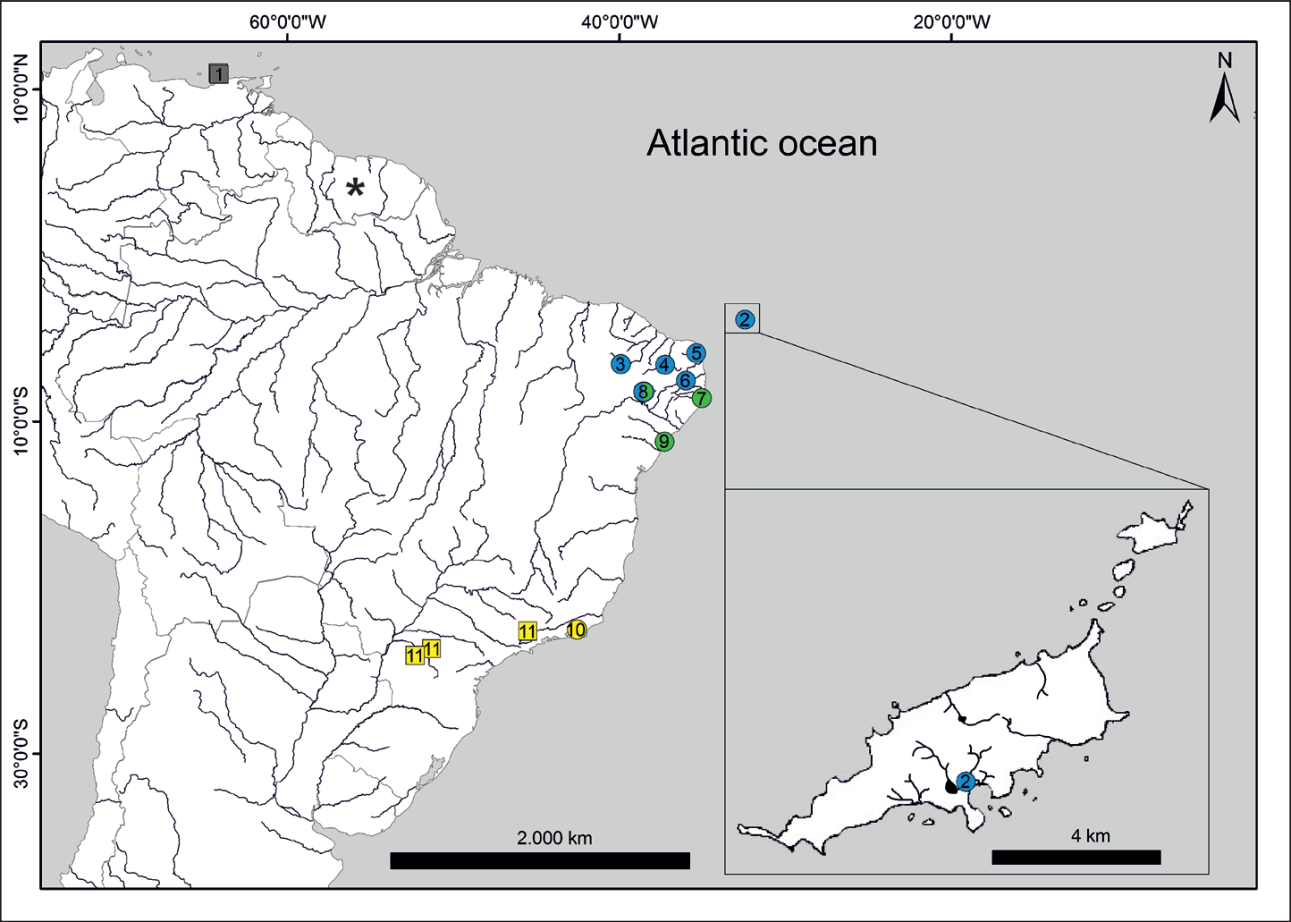
Map with the sampling sites of individuals used on genetic analyses. Circles represent sampled sites. Squares represent sequences retrieved from GenBank. An asterisk represents the type locality of *Poecilia vivipara*. Different colours represent different phylogenetic clades. Sampling sites: **1** Margarita island **2** Maceió **3** Jaguaribe **4** Piranhas-Açu **5** Potengi **6** Paraíba do Norte **7** Ipojuca **8** São Francisco **9** Piauí **10** São João **11** Paraná.

In order to determine how *P. vivipara* collected from Fernando de Noronha relate to their continental conspecifics, specimens from eight basins across northeast Brazil (including the closest coastal drainages to Fernando de Noronha archipelago), two basins from south and southwest Brazil, and one continental island in Venezuela (Isla Margarita), were included in the phylogenetic analysis (Fig. 3; Table 1). Original sequences were edited using Geneious version 6.1 software (http://www.geneious.com/), imported into MEGA v. 5.1 (Tamura et al. 2011), and aligned using the ClustalW algorithm. A total of 30 *P. vivipara* sequences were obtained. Nine *P. vivipara* sequences were retrieved from GenBank. A final alignment of 50 sequences with 610bp (including 11 sequences as outgroups) were used for the molecular analysis (Table 1).

Phylogenetic analysis was carried out using only the haplotype data (one representative per haplotype) via Bayesian Coalescent constant size tree reconstruction using Beast v.1.75 (Drummond et al. 2012). The HKY85 + G was used as the substitution model, as defined by the AIC criterion in Modeltest v. 3.7 software (Posada and Crandall 1998). A total of 10^6^ MCMC runs was performed, saving one tree every 1000 runs, resulting in a total of 1000 trees. The MCMC parameters were checked using Tracer v. 1.6 (Rambaut et al. 2014). The first 15 % of the trees were removed in order to account for the burn-in period of the analysis. A consensus tree accessing the posteriori probability values of each clade was constructed using TreeAnnotator v1.6.1 (Drummond et al. 2012).

To visualize the relationships among intraspecific haplotypes, a haplotype network was generated using PopART v. 1.7 (Leigh and Bryant 2015) with a 95 % statistical probability that multiple mutation had not occurred. Additionally, a pairwise matrix of K2P genetic distances between sampling localities was performed in order to enable comparison of the intraspecific genetic distance between and within basins. This analysis was conducted in MEGA v. 5.1.

## Results

**Figure 4.**
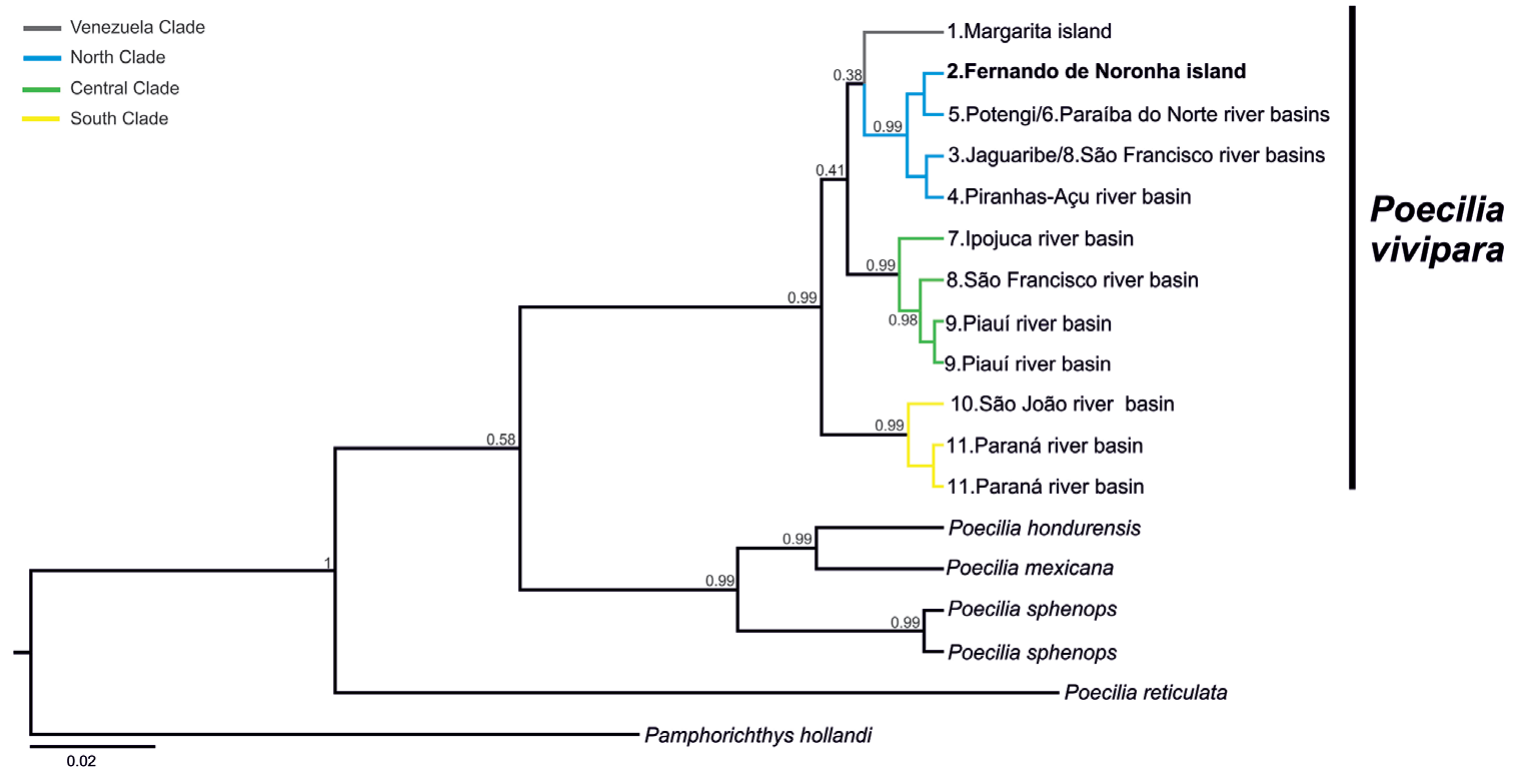
Rooted Bayesian phylogenetic reconstruction tree of Cytochrome Oxidase I mitochondrial gene of *Poecilia vivipara*. Number in nodes represents the value of posterior probability.

**Figure 5.**
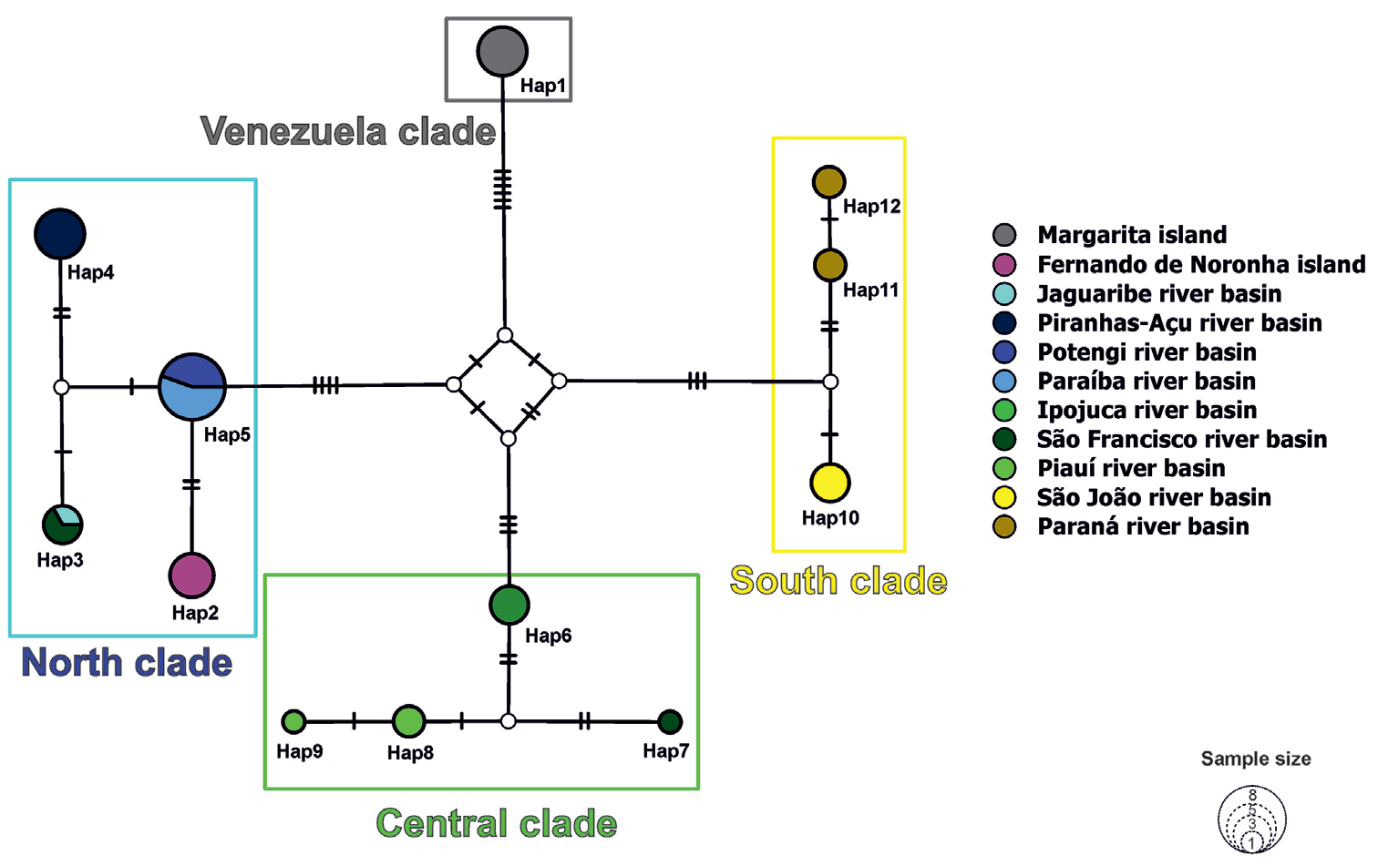
Haplotype network showing intraspecific relationships among *Poecilia vivipara* haplotypes. Empty circles represent non-sampled haplotypes. Thin bars on branches represent mutational steps.

The phylogenetic reconstruction revealed 12 haplotypes in *P. vivipara*. In most cases, each haplotype was restricted to a single drainage. In fact, only three out of eleven drainages sampled (Paraná, Piauí, and São Francisco) presented more than one haplotype. Four major clades were revealed in the phylogenetic analysis, hereafter named the ‘Venezuela’, ‘north’, ‘central’, and ‘south’ clades. The Venezuelan specimens represented one haplotype which was closely related to the samples from northeast Brazil, although with low posterior probability. The Fernando de Noronha individuals exhibited a unique haplotype, which was closely related to the shared haplotype found in Potengi and Paraíba do Norte drainages. Thus, the insular oceanic individuals fell within the ‘north clade’, comprised of lineages of northeast Brazil (Jaguaribe, Piranhas-Açu, Potengi, Paraíba do Norte, and the haplotype 3 from São Francisco). The ‘central clade’ was composed of haplotypes south of Paraíba do Norte river basin (Ipojuca and Piauí), including the São Francisco river basin (haplotype 7), located in northeast Brazil. Haplotypes found within the southernmost drainages comprised the ‘south clade’ (São João and Paraná basins) (Figs 3–5).

Our haplotype network reconstruction also showed a clear geographic pattern, within the Brazilian clades: north, central, and south clades grouping within haplogroups. The only exceptions to this pattern were the haplotypes from the São Francisco river basin. Two distantly related haplotypes (3 and 7) were found at the same sampling site; however, haplotype 3 fell within the north clade, while haplotype 7 fell within the central clade. The Fernando de Noronha haplotype is placed within the north clade, separated by two mutational steps from the haplotype shared between the Potengi and Paraíba do Norte rivers (Fig. 5).

The phylogenetic reconstruction and haplotype network clearly showed that lineage distribution followed a geographic pattern, and this pattern was corroborated by the genetic distance among drainages. The pairwise K2P distances were zero among drainages with shared haplotypes (Piranhas-Açu and Paraíba do Norte, and Jaguaribe and São Francisco), while the furthest apart sites exhibited higher genetic distance (2.6 % between Piranhas-Açu and Paraná River basins) (Fig. 4; Table 2). When compared among clades, the lowest K2P distance (1.9 %) was found between the clades that were geographically closer (north and central). The highest distance (2.2 %) was found between the most distant clades (north and south).

**Table 2.** K2P distance of *Poecilia vivipara* among basins (lower diagonal), and within basins in bold (main diagonal). River basins are MAR = Isla Margarita; MAC= Maceió; JAG= Jaguaribe; PAC= Piranhas-Açu; POT= Potengi; PBN= Paraíba do Norte; IPO= Ipojuca; SFR= São Francisco; PIA= Piauí; SAO= São João; PAR= Paraná.

	1	2	3	4	5	6	7	8	9	10	11
1.MAR	**0**										
2.MAC	0.022	**0**									
3.JAG	0.022	0.007	–								
4.PAC	0.023	0.008	0.005	**0**							
5.POT	0.018	0.003	0.003	0.005	**0**						
6.PBN	0.018	0.003	0.003	0.005	0	**0**					
7.IPO	0.018	0.017	0.017	0.018	0.013	0.013	**0**				
8.SFR	0.023	0.012	0.007	0.012	0.009	0.009	0.013	**0.013**			
9.PIA	0.024	0.022	0.022	0.024	0.019	0.019	0.005	0.017	**0.001**		
10.SAO	0.018	0.022	0.022	0.023	0.018	0.018	0.015	0.022	0.021	**0**	
11.PAR	0.021	0.024	0.024	0.026	0.021	0.021	0.017	0.024	0.021	0.006	**0.001**

## Discussion

Darwin (1859) argued that oceanic islands are most likely to be colonized by individuals from a neighbouring continent. Our results suggest that *Poecilia vivipara* from Fernando de Noronha may have derived from a genetic lineage that is closely related to the current lineage present at the closest continental drainages (north Clade, Figs 4–5). This suggests that a natural dispersal event or human assisted introduction may have occurred by fish originating from this clade. The oceanic island lineage is closely related to the shared haplotype from the Potengi and Paraíba do Norte River basins, which are geographically the closest drainages to Fernando de Noronha. The low genetic (0.3 %) differentiation observed between *P. vivipara* individuals from Fernando de Noronha and those from Potengi/Paraíba do Norte (two mutational steps) suggests that, if natural, this colonisation occurred relatively recently. Despite evidence of an exclusive lineage within the north clade, we cannot rule out the possibility that the oceanic haplotype could be originated from a non-sampled in the Rio Grande do Norte and Paraíba coastal basins. Indeed, we acknowledge that subsampling and small sample sizes are the two most common limitations when investigating source populations of biological invasions (Muirehad et al. 2008). Therefore, there are still two possible explanations for the presence of *P. vivipara* in Fernando de Noronha archipelago: human-mediated introduction and natural dispersal. The former may have occurred during United States (U. S.) military occupation of the area and after the World War II (Soto 2001). During this period of time the introduction of poeciliids was recommended to limit the spread tropical epidemic diseases by controlling mosquito larvae populations on the archipelago (Soto 2009). The closest major U. S. military base to Fernando de Noronha was located in Natal, the capital of Rio Grande do Norte State, which is by Potengi River basin (Calkins 2011). Since the 1940s, excessive amounts of DDT were spread across Fernando de Noronha island to avoid mosquito-transmitted diseases. Chemical control in addition to the intentional introduction of *P. vivipara* may have been used to control mosquito larvae population in freshwater reservoirs such as the Xaréu Reservoir on Maceió River (Serafini et al. 2010). However, the possibility of natural colonization cannot be completely ruled out.

Dispersal routes across marine barriers and the subsequent colonization events of oceanic islands by unlikely organisms have been extensively described using DNA-based methods (de Queiroz 2005). According to this author, oceanic dispersal might occur at a higher frequency or across greater distances than expected. The two major cyprinodontiform families, Cynolebiidae (previously Rivulidae) and Poeciliidae, are considered secondary freshwater fishes that are able to support and disperse through saltwater barriers (Bianco and Nordlie 2008; Albert and Reis 2011), and there is extensive evidence of saltwater dispersal among cyprinodontiform fishes, including colonization of oceanic islands (Jowers et al. 2007; de León et al. 2014).

Several factors can facilitate marine dispersal of freshwater species during particular stages of life (eggs, larvae or adults), including transport by birds, storms, rafting, ocean currents, sea level fluctuations, and decreases in superficial water salinity (Darwin 1859; Jowers et al. 2007; Measey et al. 2007). Like the vast majority of poeciliids, *P. vivipara* undertakes internal fertilization. As suggested by its specific epithet, *P. vivipara* is also viviparous, meaning offspring are released during the juvenile phase (Thibault and Schultz 1978). This removes the possibility of egg dispersal via birds or floating rafts. Storms are also an unlikely dispersal agent as they typically maintain strength for short distances (Measey et al. 2007). The ocean current passing through the Fernando de Noronha archipelago moves towards the coastal line, although some studies report drastic changes in current direction during the Pleistocene (Lumpkin and Garzoli 2005; Nunes and Norris 2006; Ludt and Rocha 2015). These historic changes in the oceanic current are corroborated by the presence of an endemic worm lizard, *Amphisbaena ridleyi* Boulenger, 1890, in the Fernando de Noronha archipelago, which is closely related to the South American group (Laguna et al. 2010); suggesting a passive natural colonization in a direction reverse to the present current direction. However, there is no record of freshwater fishes being carried by rafting (Thiel and Gutow 2005). Although *P. vivipara* is a salt-tolerant species (Gomes-Jr and Monteiro 2007), our molecular data suggests population structuring amongst continental drainages, indicating a low capacity for long-distance dispersal across continental drainages during sea level fluctuation events. Therefore, the distance between the Brazilian coast and the Fernando de Noronha archipelago is likely to be too far for a small fish such as *P. vivipara*.

Although it was not the main aim of the study, our results revealed a deep genetic structure within on *P. vivipara*. Usually, K2P distances above 2 % for COI have been considered as high intraspecific divergences, or a threshold value for species delimitation in freshwater fishes (Pereira et al. 2013). Recent studies using morphological (Lucinda 2008) and multi-locus phylogenetic data (Bagley et al. 2015) have revealed cryptic lineages within putative poeciliid species with broad geographic distribution. Our analysis revealed values above over 2 %, specifically between the lineage from Venezuela and the other clades, as well as between north and south clades As Venezuela is the geographically closest sampled site to Suriname (type locality) included herein, these high K2P distances suggest potential cryptic species of *P. vivipara* in Brazil. The high number of nominal species under the synonymy of *P. vivipara* (see Eschmeyer and Fong 2017) and its wide distribution, ranging from Venezuela to Uruguay coastal water habitats, support the possibility that *P. vivipara* may be a species complex. A further multi-locus phylogeographic study is required in order to test the evolutionary and taxonomic cohesiveness of *P. vivipara* along its wide geographic distribution.

## Conclusions

The present study represents a preliminary phylogeographical survey of *Poecilia vivipara*, a widely distributed South American guppy. Particularly, looking into the insular population of the Fernando de Noronha oceanic archipelago, which has been controversially reported as an introduced species to the archipelago. Our phylogenetic reconstruction showed a clear geographical arrangement within the distribution of *P. vivipara* lineages, and deep genetic divergence among clades. These findings indicate *P. vivipara* as a potential species complex; however, this possibility requires further investigation. The Fernando de Noronha population possibly represents an exclusive lineage which is phylogenetically related to the closest continental river basins. *Poecilia vivipara* may have been intentionally introduced into the archipelago for the purpose of mosquito larvae control during the occupation of a U.S. military base. However, given the euryhaline capacity of *P. vivipara*, a potential scenario of natural (passive or active) dispersal scenario cannot be completely disregarded. Although the origin of the archipelago lineage is still uncertain, this population may represent an interesting biological system for studies on biogeography, ecology, and evolution of isolated populations.
